# P-1462. Utilization of a Point-of-Care Hepatitis C Test to Treat in People Experiencing Homelessness with Street Medicine Outreach

**DOI:** 10.1093/ofid/ofae631.1634

**Published:** 2025-01-29

**Authors:** Yasmeen Mann, Ugonna K Ogbenna, Angela Ishak, Lauren Kasmikha, Mariia Numi, Hari Iyer, Sheema Rehman, Ali Kadouh, Ruchi Mangal, Austin Qasawa, Ali Dehghani, Muneer Hasso, Joanna Henry, Maria Santana-Garcés, Najibah K Rehman, Richard Bryce, Marcus Zervos, Seema Joshi

**Affiliations:** Henry Ford Hospital, Detroit, Michigan; Michigan State University College of Osteopathic Medicine, Southfield, Michigan; Henry Ford Hospital, Detroit, Michigan; Wayne State University School of Medicine, Detroit, Michigan; Henry Ford Hospital, Detroit, Michigan; Henry Ford Hospital, Detroit, Michigan; Henry Ford Hospital, Detroit, Michigan; Wayne State University School of Medicine, Detroit, Michigan, USA, Detroit, Michigan; Wayne State University School of Medicine, Detroit, Michigan, USA, Detroit, Michigan; Wayne State University School of Medicine, Detroit, Michigan, USA, Detroit, Michigan; Michigan State University College of Osteopathic Medicine, Southfield, Michigan; Wayne State University School of Medicine, Detroit, Michigan, USA, Detroit, Michigan; Michigan State University College of Osteopathic Medicine, Southfield, Michigan; Henry Ford Health, Livonia, Michigan; Henry Ford Health System, Detroit, Michigan; Henry Ford Hospital, Detroit, Michigan; Henry Ford Hospital, Detroit, Michigan; Henry Ford Hospital, Detroit, Michigan

## Abstract

**Background:**

Persons experiencing homelessness (PEH) have disproportionately higher rates of untreated chronic hepatitis C virus (HCV) than the public. Improved community funding and resources are needed to expand HCV rapid point-of-care (POC), confirmatory testing, treatment and follow-up. Given that PEH face competing priorities (unstable housing, food access, addiction, other infections), it is our role as clinicians to reduce barriers to HCV treatment, understand hesitancies for deferring treatment, and design programs to improve accessibility. This is the pilot project of a Street Medicine (SM) based initiative to use POC testing for HCV amongst PEH with linkage of care to a federally qualified health clinic (FQHC).

Hepatitis C Screening and Treatment Protocol

**Figure:**
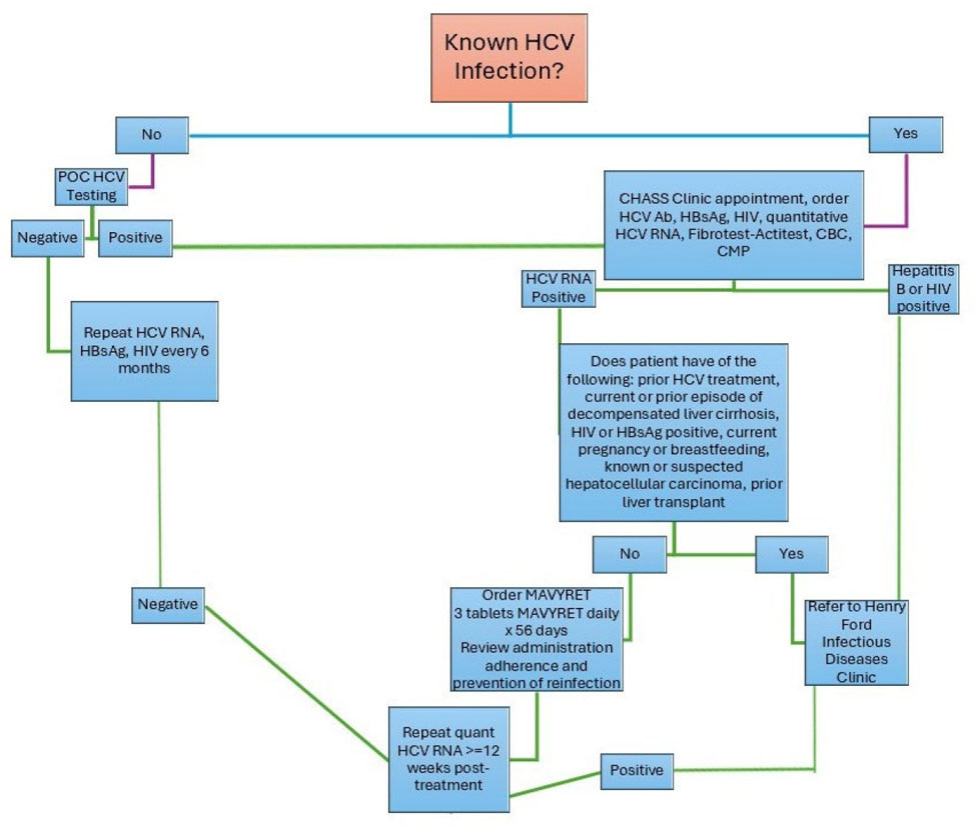

HCV- Hepatitis C Virus

POC- Point of Care

CHASS- Community Health and Social Services

HbsAg- Hepatitis B Surface Antigen

CBC- Complete Blood Count

CMP- Complete Metabolic Panel

**Methods:**

SM provides medical care to PEH outside of a hospital setting. Medical students and residents were recruited to form a HCV outreach team at Henry Ford Hospital and trained in HCV testing via OraQuick rapid antibody (Ab) test. During SM outreach, PEH were provided education on HCV and offered POC testing with a $10.00 grocery gift card. If a patient was identified as HCV Ab positive they were provided free transportation to a FQHC for confirmatory testing. If diagnosis was confirmed, they were evaluated for eligibility for simplified HCV treatment, check-ins to assess adherence and sustained virologic response (Figure 1).

**Results:**

From February-April 2024, 103 PEH were tested using POC HCV testing on SM runs, with 4 positive results. 3 of 4 patients were confirmed for CHASS clinic appointments. 1 patient presented to clinic follow-up but deferred confirmatory testing bloodwork, demonstrating challenges in arranging for HCV clinic follow-up care.

**Conclusion:**

Previous studies have shown that lack of insurance, prior authorization and referral process, active injection drug use or alcohol use, lack of knowledge of HCV treatment, and limited healthcare workforce and infrastructure have all contributed to the low treatment rate in the homeless. Using trusted community organizations with SM, this initiative aims to reduce testing and transportation barriers in order to eliminate HCV in PEH. Larger numbers of positive patients are needed to best assess the feasibility of the program, and if providing all testing and treatment at the point of care is needed to improve compliance.

**Disclosures:**

**Marcus Zervos, MD**, Johnson and johnson: Grant/Research Support|Moderna: Grant/Research Support **Seema Joshi, MD**, AbbVie: Grant/Research Support

